# RETRACTED: Atta et al. Preparation and Application of Crosslinked Poly(sodium acrylate)-Coated Magnetite Nanoparticles as Corrosion Inhibitors for Carbon Steel Alloy. *Molecules* 2015, *20*, 1244–1261

**DOI:** 10.3390/molecules31142494

**Published:** 2026-07-17

**Authors:** Ayman M. Atta, Gamal A. El-Mahdy, Hamad A. Al-Lohedan, Ashraf M. El-Saeed

**Affiliations:** 1 Surfactants Research Chair, Department of Chemistry, College of Science, King Saud University, P.O. Box 2455, Riyadh 11451, Saudi Arabia; gamalmah2000@yahoo.com (G.A.E.-M.); lohedan@ksu.edu.sa (H.A.A.-L.); 2 Petroleum Application Department, Egyptian Petroleum Research Institute, Cairo 11727, Egypt; ashrfelsaied@yahoo.com; 3 Chemistry Department, Faculty of Science, Helwan University, Helwan, Cairo 11795, Egypt

The Journal retracts the article “Preparation and Application of Crosslinked Poly(sodium acrylate)-Coated Magnetite Nanoparticles as Corrosion Inhibitors for Carbon Steel Alloy” [1] cited above.

Following publication, concerns were brought to the attention of the Editorial Office regarding the reliability of a figure presented in this article [1].

In accordance with standard journal procedures, the Editorial Office, in consultation with the Editorial Board, conducted an investigation. This investigation confirmed overlaps between images in Figure 2 of this publication [1] and figures published in five other articles [2,3,4,5,6], produced by partially overlapping author groups and reported under different experimental conditions. Although the authors cooperated with the investigation, the explanations and supporting materials provided were not deemed sufficient by the Editorial Board to resolve the identified concerns. As a consequence, the Editorial Board has lost confidence in the reliability of the reported findings and has decided to retract this publication in accordance with MDPI’s retraction policy.

This retraction was approved by the Editor-in-Chief of the journal *Molecules.*

The authors have been informed of this retraction but did not provide a comment on this decision.
